# Azithromycin as macrolide of choice in patients with preterm premature rupture of membranes in expectant management

**DOI:** 10.61622/rbgo/2026rbgo38

**Published:** 2026-09-23

**Authors:** Fernanda Castilhos Bosi, Gabriel Cardozo Müller, Janete Vettorazzi, Daniela Vanessa Vettori, Maria Carolina Bittencourt da Costa, Mariana Sbaraini, Rafaela Rodolfo Tomazzoni, Edimárlei Gonsales Valério

**Affiliations:** 1 Hospital de Clínicas de Porto Alegre Porto Alegre RS Brazil Hospital de Clínicas de Porto Alegre, Porto Alegre, RS, Brazil.; 2 Universidade Federal do Rio Grande do Sul Department of Epidemiology Porto Alegre RS Brazil Department of Epidemiology, Universidade Federal do Rio Grande do Sul, Porto Alegre, RS, Brazil.; 3 Universidade Federal do Rio Grande do Sul School of Medicine Porto Alegre RS Brazil School of Medicine, Universidade Federal do Rio Grande do Sul, Porto Alegre, RS, Brazil.; 4 Hospital Geral de Caxias do Sul Caxias do Sul RS Brazil Hospital Geral de Caxias do Sul, Caxias do Sul, RS, Brazil.

**Keywords:** Pregnant women, Pregnancy complications, infectious, Obstetric labor, Preterm premature rupture of the membranes, Azithromycin, Amoxicillin, Ampicillin, Anti-bacterial agents, Birth rate, Infant newborn

## Abstract

**Objective:**

This study seeks to demonstrate that the use of antibiotics, with azithromycin as macrolide of choice, in expectant management reduces the birth rate in the first 48 hours after PPROM, increasing the latency period between PPROM and birth.

**Methods:**

Observational, retrospective cohort study in pregnant women with PPROM treated conservatively at the Hospital de Clínicas de Porto Alegre, from January 2012 to December 2019. For the group that used antibiotics, the scheme was Azithromycin and Ampicillin for 48 hours, followed by Amoxicillin for 5 days, completing 7 days of treatment.

**Results:**

145 participants, of which 107 did not use antibiotics (Group 1) and 38 used antibiotics (Group 2). The 48-hour birth rate was significantly lower in the antibiotics group (33.6% in Group 1 *vs.* 16% in Group 2, p=0.04). The birth rate on patients presenting outcomes before 15 days presented a significant reduction (HR 0.6 [95% CI 0.39-0.92]), as well median time to birth as seen in Kaplan-Meier estimator by log-rank test.

**Conclusion:**

The use of this antibiotic's regimen, with azithromycin as macrolide of choice, reduces delivery within 48 hours, with potential clinical benefits due to the increase in the pharmacological window for corticosteroid action, among others, and increased median time to birth in first 15 days between PPROM and birth.

## Introduction

Premature rupture of membranes (PROM) is when spontaneous rupture of fetal membranes occurs before labor. Preterm PROM (PPROM) is when this event happens before 37 weeks of gestation.^(1)^ PPROM is an important cause of prematurity that occurs in 3% of all deliveries, and is associated with 1/3 of all premature births.^(2,3)^ On the other hand, prematurity is associated with 35% of neonatal deaths, being an important public health marker.^(4)^ Lower gestational age and small birth weight are factors that are greatly associated with increased child mortality rate.^(5)^

Management of patients with PPROM is still controversial, and there is no clear evidence on the ideal gestational age for termination of pregnancy. Nevertheless, expectant management between gestational ages (GAs) from 23-24 weeks to 33 weeks + 6 days is currently recommended. Between GAs of 34 to 36 weeks + 6 days, ACOG considers possible both immediate interruption and expectant management, but there is no consensus regarding this conduct among the main guidelines around the world.^(1)^ Immediate interruption is indicated for GAs above 37 weeks, when there are signs of maternal and/or fetal infection, or non-reassuring fetal status. The interruption is performed, in most cases, with induction of labor - if there is no contraindication to vaginal delivery.^(6)^

The etiology of PPROM is multifactorial and often idiopathic. Infection can be associated both as a cause and as a consequence; therefore, in cases of expectant management, the use of antibiotics is widely investigated. The routine use of antibiotics seems to decrease maternal and fetal morbidity, in addition to increasing the latency period between rupture of membranes and childbirth. Antibiotics reduce microbial load and subclinical inflammation, both of which precipitate preterm labor and increase the risk of maternal and neonatal infections. Their use prolongs the latency period, allowing for interventions such as antenatal corticosteroid administration and fetal neuroprotection, which significantly improve perinatal outcomes.^(7)^ The ideal combination of types of antibiotics and dosage is not yet well established and needs further studies to be elucidated.^(8)^ The regimen recommended by ACOG is of erythromycin combined with ampicillin and amoxicillin.^(1)^ However, other antibiotics regimens have been studied, showing similar outcomes.^(9-11)^

The primary objective of this study is to evaluate the effectiveness of using Azithromycin and Ampicillin, followed by Amoxicillin, in reducing the birth rate in the first 48 hours after PPROM and increasing the latency time between PPROM and childbirth. Secondary objectives include evidence of a reduction in rates of chorioamnionitis, endometritis, perinatal morbimortality, among others.

## Methods

Observational, retrospective cohort study in pregnant women with PPROM treated expectantly at Hospital de Clínicas de Porto Alegre, Rio Grande do Sul - Brazil, from January 2012 to December 2019. We consecutively included all women admitted to our institution with a diagnosis of PPROM, with gestational ages between 23 weeks and 33 weeks and 6 days, between 2012 and 2019 who met the predefined inclusion and exclusion criteria. Patients were identified through the mandatory ICD admission diagnosis code, which is systematically recorded at hospital entry. Each medical record was reviewed in full, and no eligible cases were excluded due to missing charts or insufficient information regarding exposures or outcomes. The detailed selection process is presented in the flowchart (Figure 1). Women who were already using antibiotics at the time of PPROM and/or had used any antibiotics in the last 7 days were excluded. Fetuses weighing less than the 10th percentile at admission and/or with malformations were excluded since they were potential confounding factors in the analysis of neonatal data. Pregnant women with cervical cerclage, uterine malformations and/or multiple pregnancies were also excluded.

**Figure 1:**
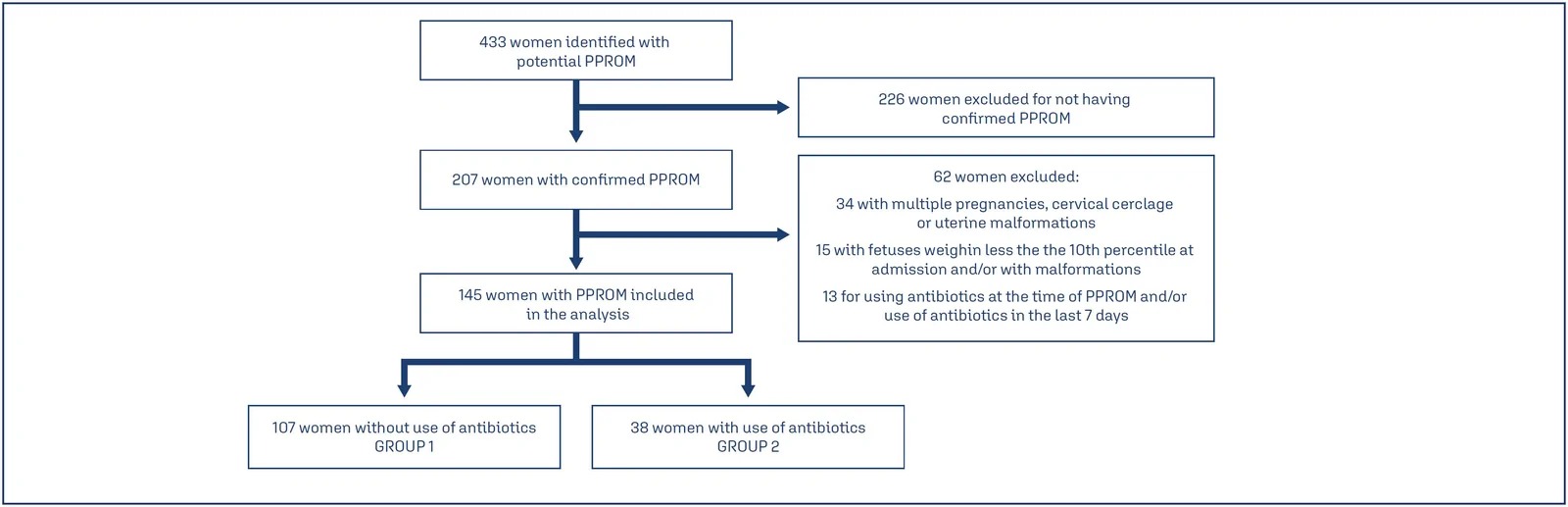
Flowchart of patient selection with PPROM

Participants were divided into two groups: Group 1 did not receive any antibiotics and Group 2 received antibiotics. The regimen used in Group 2 was oral Azithromycin 1 gram (g) single dose with intravenous Ampicillin 2 g as the loading dose and 1 g every 6 hours for 48 hours as the maintenance dose, followed by Amoxicillin 500 mg every 8 hours for 5 days - completing 7 days of treatment. Latency time was defined as the time between PPROM and birth. The decision to administer antibiotics was based on individual team protocols within Hospital de Clínicas de Porto Alegre, as well as on chronological factors, given that after 2017, a standardized protocol was introduced recommending that all teams administer antibiotics to these patients.

The diagnosis of amniorrhexis was made by specular examination, and gestational age was calculated using the earliest ultrasound performed. Participants were followed according to hospital protocol. Group B streptococcus (GBS) was investigated with vaginal and rectal swab, except for those already considered colonized, with urinary infection by GBS in this pregnancy or with a previous child who had GBS infection. In both groups, women with preterm PPROM received intrapartum antibiotic prophylaxis only when colonized with GBS. The ‘no-antibiotic’ group refers solely to the absence of antibiotic therapy for PPROM; however, intrapartum prophylaxis could still be administered when clinically indicated. Fetal wellbeing was assessed with fetal biophysical profile and serial cardiotocography. Ultrasound and tests such as blood count and urine analysis were also serially performed to assess fetal growth and to investigate infection, respectively. Data were collected for all participants in the period between January 2012 and December 2019.

The expected difference in birth rate in the first 48 hours after PPROM was of 32% between the group patients that received intrapartum antibiotic prophylaxis and without this intervention, using estimates obtained from Grable et al.^(12)^ We calculated that enrollment of 108 participants (27 in the first group and 81 in the second group) would provide a power of 90% to detect this difference, with an alpha of 0.05. A great variability of this rate is found in the literature and is altered with the type of primary analyses that were made; however, we considered that this sample size was the minimum necessary to find a statistically significant difference considering a large effect size.

Analyses were performed using R Statistical Software (version 4.0.0, R Core Team. R: A language and environment for statistical computing. R Foundation for Statistical Computing, Vienna, Austria) and RStudio (version 1.1.4 RStudio Team. RStudio: Integrated Development for R. RStudio, Inc., Boston, MA). Numerical variables were evaluated for normality by histograms and by the Shapiro-Wilk test, and, presenting asymmetry in these distributions, Mann-Whitney test was used. To dichotomous outcomes, Chi Square or Fisher exact test were used, and we also estimated Relative Risk and 95% confidence intervals, using 5000 bootstrap simulations, as suggested by Mwangi et al.^(13)^ for unbalanced groups. Time-to-event analysis was performed using Kaplan-Meier estimator, with comparison among groups made by log-rank test.

Cox Proportional Hazards regression was used to control for confounders, estimating models as proposed by Colosimo et al.,^(14)^ checking proportional hazards assumption by visual inspection of Schoenfeld residuals and testing for trends towards time. In order to adjust for best fit model, we used likelihood ratio test, Akaike and Bayesian Information Criteria (AIC and BIC). Differences were considered as statistically significant when the p-value was less than 0.05.

The study was approved by the Research Ethics Committee of Hospital de Clínicas de Porto Alegre under CAAE number 16917619.3.0000.5327 and approval number 4.435.722. Given the retrospective design and the use of anonymized data, the need for informed consent was waived in accordance with national regulations.

## Results

From January 2012 through December 2019, 433 potential participants were identified. Among these, 226 were excluded for not having criteria for PPROM or having gestational age less than 23 weeks or more than 34 weeks. The remaining 207 women had a diagnosis of PPROM and expectant management was indicated, but 62 had potential confusion factors for the outcomes and were excluded (Figure 1). A total of 145 pregnant women were included: 107 did not receive antibiotics (Group 1) and 38 received antibiotics (Group 2). Baseline characteristics of each group with information obtained on hospital admission (Table 1), showing similarity between the groups.

**Table 1:** Obstetric and demographic data of the women included in the study

Demographic data at admission		Group 1 (No antibiotics)	Group 2 (With antibiotics)
n		107	38
Age, Years		26.0[21.0,33.0]	25.0[20.0,33.8]
Level of schooling			
	Complete primary school	20(18.7)	7(18.4)
	Incomplete elementary school	40(37.4)	11(28.9)
	Complete high school	26(24.3)	11(28.9)
	Incomplete high school	9(8.4)	4(10.5)
	Complete higher education	3(2.8)	1(2.6)
	Incomplete higher education	9(8.4)	4(10.5)
Race			
	White	71(66.4)	26(68.4)
	Black	20(18.7)	9(23.7)
	Mixed	16(15.0)	3(7.9)
Number of prenatal care visits		4.0[3.0,6.0]	5.0[3.0,6.0]
Parity			
Nulliparous		37(34.6)	12(31.6)
Gestational age at PPROM			
	23 weeks to 27 weeks + 6 days	21(19.6)	10(26.3)
	28 weeks to 30 weeks + 6 days	21(19.6)	5(13.1)
	31 weeks to 33 weeks + 6 days	65(60.7)	23(60.5)
Maternal comorbidities			
	Gestational diabetes	6(4.3)	9(15.8)
	Chronic hypertension	4(2.8)	1(1.8)
	Urinary tract infection during this pregnancy	26(18.4)	14(24.6)
	Previous premature labor	18(12.8)	3(5.3)
	Inhibition of premature labor during this pregnancy	9(6.4)	1(1.8)
	Previous PPROM	7(5.0)	1(1.8)
	Smoking	23(16.3)	5(8.8)
	Asthma	4(2.8)	5(8.8)
	Psychiatric disorders	7(5.0)	1(1.8)
	First trimester bleeding	6(4.3)	0(0.0)
	Other maternal pathologies	31(22.0)	17(29.8)

Latency period: The 48-hour birth rate was significantly lower in the antibiotics group (n=36 [33.6%] in Group 1, *vs.* 6 [16%] in Group 2, RR 0.46 [95% CI 0.15-0.90], p=0.04), as shown in table 2. The 7-day birth rate was also lower in the antibiotics group (n=68 [63.5%] in Group 1 *vs.* 18 [47%] in Group 2, RR 0.74 [95% CI 0.47-1.03], p=0.09), though without statistical significance.

**Table 2:** Obstetrics outcomes and maternofetal complications

		Group 1 (No antibiotics)	Group 2 (With antibiotics)	p-value	RR	95% CI
n		107	38			
Outcomes						
Birth in the first 48 hours		36(33.6)	6(15.8)	0.04	0.46	0.15-0.90
Birth in the first 7 days		68(63.6)	18(47.4)	0.09	0.74	0.47-1.03
1-minute APGAR < 7		32(29.9)	8(21.1)	0.39	0.70	0.28-1.28
5-minutes APGAR < 7		8(7.5)	3(7.9)	1.00	1.05	0.00-3.51
Birthweight. grams		1935[1503.8, 2133.8]	1900[1507.5, 2142.5]	0.82	-	-
Arterial cord pH		7.31[7.26,7.35]	7.29[7.25, 7.34]	0.62	-	-
Arterial cord base excess		-3[-5.5,-1.8]	-4.2[-6.1, -2.2]	0.25	-	-
ICU length of stay, days		20[11.0,43.0]	21[14.0, 33.25]	0.82	-	-
Neonatal complications						
	Surfactant use	24(22.4)	10(26.3)	0.65	1.17	0.56-2.17
	Neonatal ICU hospitalization	104(97.2)	37(97.4)	1.00	1.00	0.92-1.05
	Ventilatory support	79(73.8)	30(78.9)	0.66	1.06	0.85-1.29
	Necrotizing enterocolitis	10(9.3)	2(5.3)	0.73	0.56	0.00-1.87
	Presumed neonatal sepsis	82(76.6)	27(71.1)	0.52	0.93	0.72-1.15
	Intracranial hemorrhage	11(10.3)	6(15.8)	0.38	1.54	0.43-3.75
	Neonatal pneumonia	11(10.3)	0(0.0)	0.07	0.00	0.00-0.00
	Neonatal death	6(5.6)	3(7.9)	0.69	1.40	0.00-5.63
	Meningitis	6(5.6)	0(0.0)	0.34	0.00	0.00-0.00
	Cardiorrespiratory arrest	2(1.9)	2(5.3)	0.28	2.82	0.00-0.00
	Septic shock	5(4.7)	1(2.6)	1.00	0.56	0.00-2.81
	Other complications	15(14.0)	5(13.2)	1.00	0.93	0.21-2.19
Maternal complications						
	Clinical chorioamnionitis	28(26.2)	9(23.7)	0.83	0.90	0.41-1.66
	Endometritis	6(5.6)	0(0.0)	1.00	0.00	0.00-0.00
	Maternal sepsis	1(0.9)	0(0.0)	1.00	0.00	0.00-0.00
	Additional hypotonia management	4(3.7)	5(13.3)	0.053	3.52	0.70-22.52

We also performed time-to-event analysis using Cox regression, fitting several multivariate models. Even so, the best fit was univariate with antibiotics treatment. Also, in order to do not violate proportional hazards assumption, we stratified our population in those with event before and after 15 days.

In population with event before 15 days, we observed a significant reduction in event rate HR 0.6 [95% CI 0.39-0.92] when comparing antibiotics treatment group with non-treated group, as estimated by Cox PH. Also, by Kaplan-Meier estimator (Figure 2), we also detected higher median time in G2 (94 hours [95% CI 72-216]) in comparison with G1 (70 hours [95% CI 45-96]), as detected by log-rank test (chi-square=5.3, 1 d.f., p=0.02) (Figure 2a).

**Figure 2:**
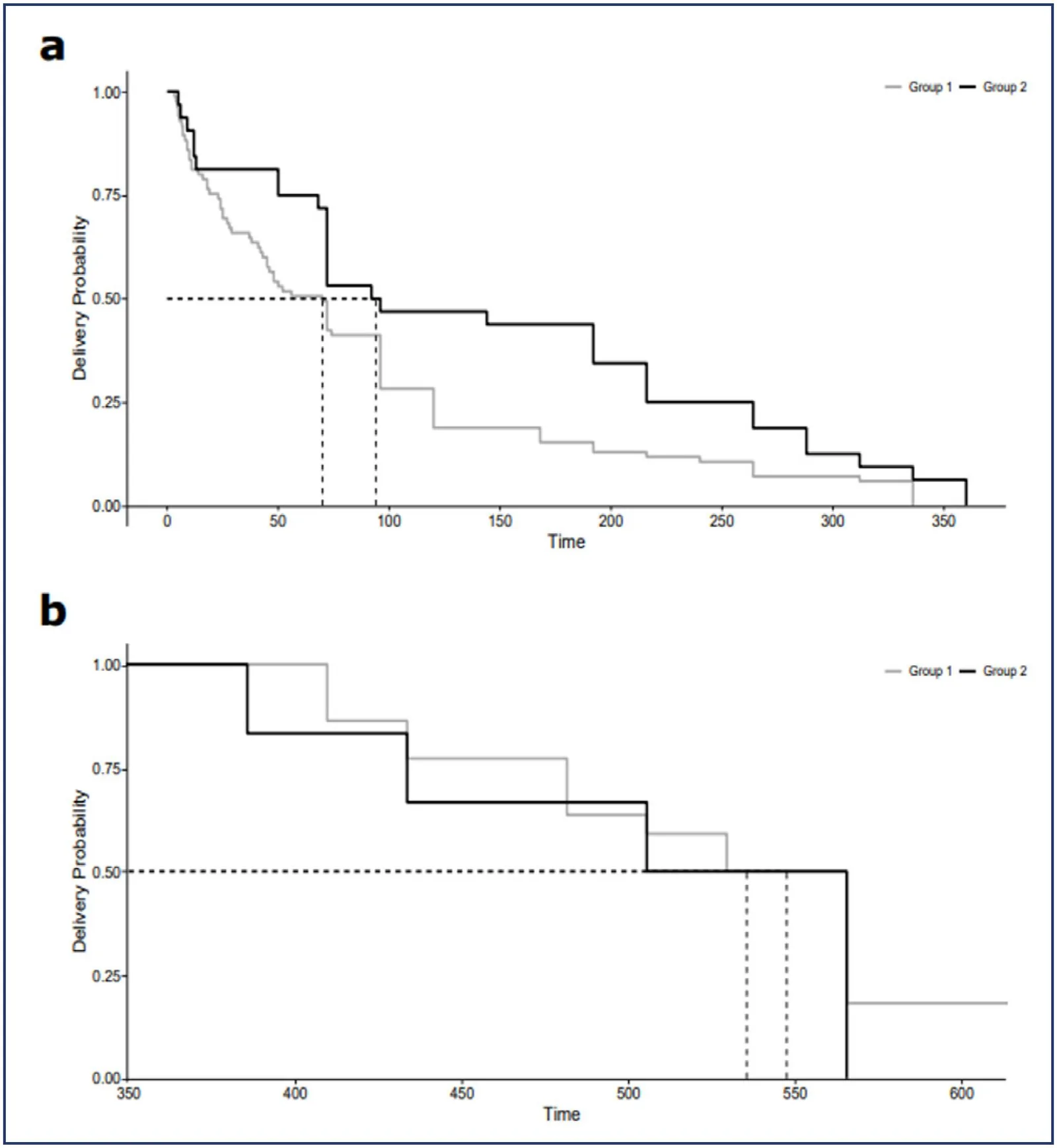
Kaplan-Meier curves comparing time to delivery between antibiotic-treated (G2) and non-treated (G1) groups

Even so, in strata of birth after 15 days, we did not observe a significant difference both in Cox (HR 2.2 [95% CI 0.76-6.39], p=0.14) model and Kaplan-Meier curve (546 hours [95% CI 450-564] in G1 and 534 hours [95% CI 432-Inf] in G2, Chi-square=0.4, 1 d.f., p=0.50, log-rank test) (Figure 2b). However, the low number of events may explain the wide variance, limiting robustness of these estimates.

Obstetrics outcomes and maternofetal complications: No significant difference between groups was found for other outcomes described in table 2, such as the need for admission to a neonatal ICU and/or days of hospitalization, APGAR in 1 and 5 minutes, pH and excess base in the newborn's blood gas analysis and birth weight. Caesarean indications were similar between groups.

We also described neonatal complications (Table 2), finding no significant difference was found between the groups. Six (5.6%) newborns with meningitis were identified in Group 1, and none in the antibiotics group. Neonatal pneumonia was present in 11 newborns in Group 1 (10.28%, p=0.19) and in 1 in Group 2 (2.56%, p=0.18). There was also no difference in the outcome of necrotizing enterocolitis (10 newborns [9.35%] in Group 1 *vs.* 2 [5.13%] in Group 2, p=0.51). As for maternal complications, endometritis was found in six postpartum women in Group 1 and none in the antibiotics group, but without statistical significance (p=0.19). There was also no significant difference between groups regarding maternal sepsis or the need for management of uterine atony. Participants in the antibiotic group required increased management for hypotonia control; however, this was not statistically significant. Anatomopathological examination of 123 placentas was performed, showing chorioamnionitis in 76 placentas, with no difference between groups.

## Discussion

Our cohort study demonstrates an increased latency time between PPROM and birth in the group that received antibiotics, reducing the 48-hour birth rate and increased median latency time in 15 days strata, as well rate of events on this specified period. Some studies show similar results, with increased latency time with the use of antibiotics.^(15,16)^ However, to our knowledge, only a few studies have evaluated the antibiotics regimen of azithromycin and ampicillin, followed by amoxicillin, in comparison with no antibiotic use.

The main international guidelines recommend the combination of a beta-lactam antibiotic (penicillin, ampicillin and/or amoxicillin) associated with a macrolide (erythromycin).^(1,17,18)^ Nevertheless, a recent meta-analysis concluded that data in this subject are still insufficient to determine the best antibiotic regimen.^(19)^ With the exception of amoxicillin + clavulanate, which increased the risk of necrotizing enterocolitis, no antibiotic regimen was found to be superior to the other.^(8)^

The combination that was administered in our study, using azithromycin as the macrolide of choice, has been tested more recently and appears to be a good option, due to having a lower cost, easier administration (single oral dose), easier access and availability in developing countries, and reduced side effects when compared to erythromycin, with no difference in latency time between groups.^(10,20-22)^ It is noteworthy that erythromycin is not available in hospitals funded by the Brazilian Unified Health System (SUS), which represents a country-specific limitation that may not be applicable to other healthcare systems.

In this context, a multicenter retrospective cohort compared different doses of azithromycin (single dose, 5 days or 7 days) versus erythromycin, showing no difference in latency time between the 4 groups. The latency time was on average 5 days, while in our study the average time was higher, of 8 days.^(22)^

Compared to a study conducted by Kenyon et al,^(23)^ we found similar 48-hour birth rates in the group without antibiotics (35.6%*vs.*33.6%, respectively); however, our study had a lower percentage of births in the first 48 hours in the group with antibiotics (33.0%*vs.*16.0%). A similar numerical difference was observed when comparing 7-day birth rates (60.5%vs.63.5% in the no-antibiotics group and 57.7%vs.47.0% in the antibiotics group), although this difference was not statistically significant.^(23)^ On time to event analysis, this antibiotic scheme has increased latency, limited to 15 days strata, that could be explained to antibiotics pharmacokinetics.^(24)^

A 2013 Cochrane review with 22 randomized clinical trials showed that the use of antibiotics in PPROM reduced the rates of chorioamnionitis, abnormal brain ultrasound before hospital discharge, neonatal infection, and surfactant and oxygen use in the newborn.^(8)^ Among the many benefits of reducing the 48-hour birth rate after PPROM, which was demonstrated in this study, is the increase of the therapeutic window for administration of antenatal corticosteroids. Its use after PPROM is associated with a reduction in neonatal mortality, respiratory dysfunction, intraventricular hemorrhage and necrotizing enterocolitis.^(7,25-27)^

Our study demonstrated lower rates of endometritis, pneumonia, and neonatal meningitis in the group that received antibiotics, although these differences did not reach statistical significance. Although these findings may suggest a higher infectious risk in the absence of antimicrobial coverage, the observed differences were not statistically significant. Given that neonatal infections represent an important cause of morbidity, need for intensive support, and increased early neonatal complications, this non-conclusive result underscores the clinical relevance of vigilant monitoring and reinforces the need for studies with greater statistical power to confirm this association. The lack of differences observed in maternal and neonatal outcomes may reflect the limited sample size, which reduces the statistical power to detect true differences, particularly for less frequent events, and constitutes a key limitation of the study. Due to the impossibility of conducting a prospective study with this design for ethical reasons, we suggest further retrospective studies with a larger sample size enrolled, in order to better evaluate maternal and neonatal outcomes. In addition, we also highlight the need to conduct randomized clinical trials comparing different antibiotic regimens, considering that there is still a gap regarding the ideal antibiotic regimen (drugs and doses) to be used in this group of patients.^(28,29)^

Another important limitation of our study is that GBS colonization was not analyzed as a variable. In our institution, intrapartum antibiotic prophylaxis is administered only to women colonized with GBS, regardless of the PPROM management strategy. Because we were unable to account for this factor, residual confounding is likely, particularly in relation to neonatal infectious outcomes. The inability to adjust for GBS status may have influenced the associations observed and should be considered when interpreting our results.

## Conclusion

In women with PPROM, the use of the antibiotics regimen consisting of oral Azithromycin 1 g single dose and intravenous Ampicillin 2 g as the loading dose and 1 g every 6 hours for 48 hours as maintenance, followed by Amoxicillin 500 mg every 8 hours for 5 days (total of 7 days of treatment), significantly reduced the 48-hour birth rate and time to birth in 15 days strata, compared to not using antibiotics. Confirming previous findings, it also increased the latency time between PPROM and birth, with an important clinical benefit by increasing the pharmacological action window for corticotherapy. However, these effects on latency did not translate into significant improvements in neonatal outcomes. We believe that the lack of statistical significance may be largely attributable to the limited sample size, which reduces the power to detect differences, particularly in less frequent outcomes. This represents an important limitation of our study. Larger, adequately powered prospective studies are needed to clarify the true clinical impact of this regimen.

## Data Availability

The research data are described in the article presented.
